# Gene-Editing in Respiratory Disease: From Pre-Clinical Human Models to Future Therapeutics

**DOI:** 10.3390/biomedicines14102237

**Published:** 2026-10-03

**Authors:** Amy Duffy, Anja Schweikert, Sze Ying Tan, Cormac Murphy, Catríona M. Dowling, Killian Hurley, Mari Ozaki

**Affiliations:** 1 Department of Medicine, Royal College of Surgeons in Ireland, Education and Research Centre, Beaumont Hospital, Dublin 9, D09 YD60 Dublin, Ireland; 2 Tissue Engineering Research Group, Royal College of Surgeons in Ireland, Dublin 2, D02 YN77 Dublin, Ireland

**Keywords:** gene-editing, genetic lung disease, gene-therapies, disease modelling, iPSCs, CRISPR

## Abstract

Chronic lung diseases arise from complex interactions between environmental exposures and host genetic factors. However, these interactions are not fully understood, and despite decades of study, effective curative therapies remain unavailable for many patients. Recent breakthroughs in therapeutic gene-editing have become a reality for some inherited blood diseases and are a promising approach for monogenic lung diseases. Early-phase clinical studies are now ongoing for AATD, primarily through liver-directed editing of hepatocytes to restore circulating functional AAT and for COVID-19 using gene-edited immune cell approaches. However, direct in vivo gene-editing of pulmonary epithelial cells remains largely at the preclinical stage. Expanding this therapeutic pipeline requires overcoming the unique biological barriers of the lung and developing human-specific models that faithfully recapitulate human disease mechanisms. Advances in gene-editing technologies, including CRISPR-Cas9 systems, base and prime editors, and emerging compact nucleases, have increased the feasibility of precise genomic correction. However, safe and efficient delivery directly to the lung remains a major unmet challenge due to mucus, surfactant, epithelial tight junctions, and innate immune surveillance. Delivery systems such as engineered AAVs, lentiviral vectors, selective organ targeting lipid nanoparticles and aerosolizable formulations like nebulised or dry powder nanoparticles are beginning to address these obstacles. In parallel, human preclinical models have evolved from traditional cell lines to more sophisticated platforms such as iPSC-derived airway and alveolar cells, organoids, precision-cut lung slices, air–liquid interface cultures, organ-on-a-chip devices, and patient-derived primary cells. These models enable mechanistic interrogation, variant-specific functional assessment, and preclinical evaluation of gene-editing strategies with increased physiological relevance. In this review, we detail current and emerging gene-editing tools, delivery strategies, and human-relevant disease models. We highlight key translational barriers and discuss how advanced human models combined with improved delivery technologies can accelerate the development of safe and effective gene-editing therapeutics for chronic respiratory disease.

## 1. Introduction

Chronic respiratory diseases (CRDs) are among the most significant global health challenges, affecting over 468.3 million individuals worldwide and resulting in 4.4 million deaths in 2021, marking a 46.6% increase in deaths since 1990 [1]. Chronic lung diseases, such as chronic obstructive pulmonary disease (COPD), pulmonary fibrosis (PF), cystic fibrosis (CF), and alpha1-antitrypsin deficiency (AATD), arise from complex interactions between environmental exposures and genetic factors, and treatments for these diseases have variable efficacy. Damage to the lung caused by these diseases are often irreversible, driving demand for lung transplantation [2], which cannot be met. Understanding the underlying mechanisms of lung tissue damage is paramount.

Recent advances in gene-editing technologies have transformed the therapeutic landscape. The approval of the first CRISPR-based therapy, Casgevy™, for sickle cell disease and β-thalassemia demonstrated that precise genome editing can be safe and clinically effective in humans [3]. This milestone was followed by the first successful Phase 3 trial of an in vivo CRISPR therapeutic, in which a lipid nanoparticle (LNP)-delivered Cas9 system targeting *KLKB1* in hepatocytes produced sustained gene inactivation and a profound reduction in hereditary angioedema attacks [4,5]. These achievements underscore the translational potential of in vivo genome editing and have catalysed efforts to extend gene editing to monogenic lung diseases. Early-phase clinical studies are currently ongoing for AATD, focusing mainly on liver-targeted editing of hepatocytes to restore functional levels of circulating AAT, as well as for COVID-19, using gene-edited immune cell strategies. However, direct in vivo gene-editing of pulmonary epithelial cells remains largely confined to the preclinical stage [6,7,8,9].

The lungs complex branching structure, mucus barrier, surfactant layer, tight epithelial junctions, and robust innate immune defences impede delivery of gene-editing tools [10]. Overcoming these barriers not only requires better delivery systems but human-specific disease models that can faithfully recapitulate lung biology and enable mechanistic insight into disease pathogenesis. Traditionally, animal models and basic human in vitro disease models, such as immortalised lung cell lines, have been used to study respiratory disease mechanisms [11,12]. More advanced cell models, including induced pluripotent stem cell (iPSC)-derived lung cells, spheroids or organoids, precision-cut lung slices (PCLS), air–liquid interface (ALI) cultures, organ-on-a-chip (OOC) devices and patient-derived primary cells now offer platforms with unprecedented physiological relevance [13,14,15,16]. These models can be used in combination with tools such as Clustered Regularly Interspaced Short Palindromic Repeats (CRISPR), prime and base editors or Peptide Nucleic Acids (PNA) to introduce disease-specific mutations. Together, these developments create a powerful opportunity to integrate advanced gene-editing tools, innovative delivery strategies, and relevant human models to identify and evaluate therapeutic responses.

In this review, we describe emerging gene-editing technologies, such as CRISPR activation (CRISPRa) and CRISPR interference (CRISPRi), and base- and prime-editing. We discuss how these tools are being adapted to address drivers of pulmonary disease and their potential application in both target discovery and emerging therapeutics. We highlight the biological barriers of the lung that make pulmonary gene-editing uniquely challenging and critically evaluate the strengths and limitations of current human preclinical models. The lung is highly heterogeneous, requiring efficient gene-editing to not only be delivered to the lung but, in some cases, to access disease-relevant cell populations. This is complicated further by surfactant, matrices, and immune cells that can rapidly clear delivered gene-editing tools. In this review, we examine emerging delivery strategies, including viral and non-viral approaches, and discuss emerging modifications to existing tools to improve lung targeting, safety, and efficiency. Finally, we highlight how advanced gene-editing tools, when paired with physiologically relevant human systems, can enable precise target discovery, mechanistic insight, and therapeutic drug screening. Together, these developments point toward a future in which safe and effective gene-editing therapeutics for chronic lung disease can be rationally designed, rigorously tested, and translated with greater confidence.

## 2. Gene-Editing Tools

**Figure 1:**
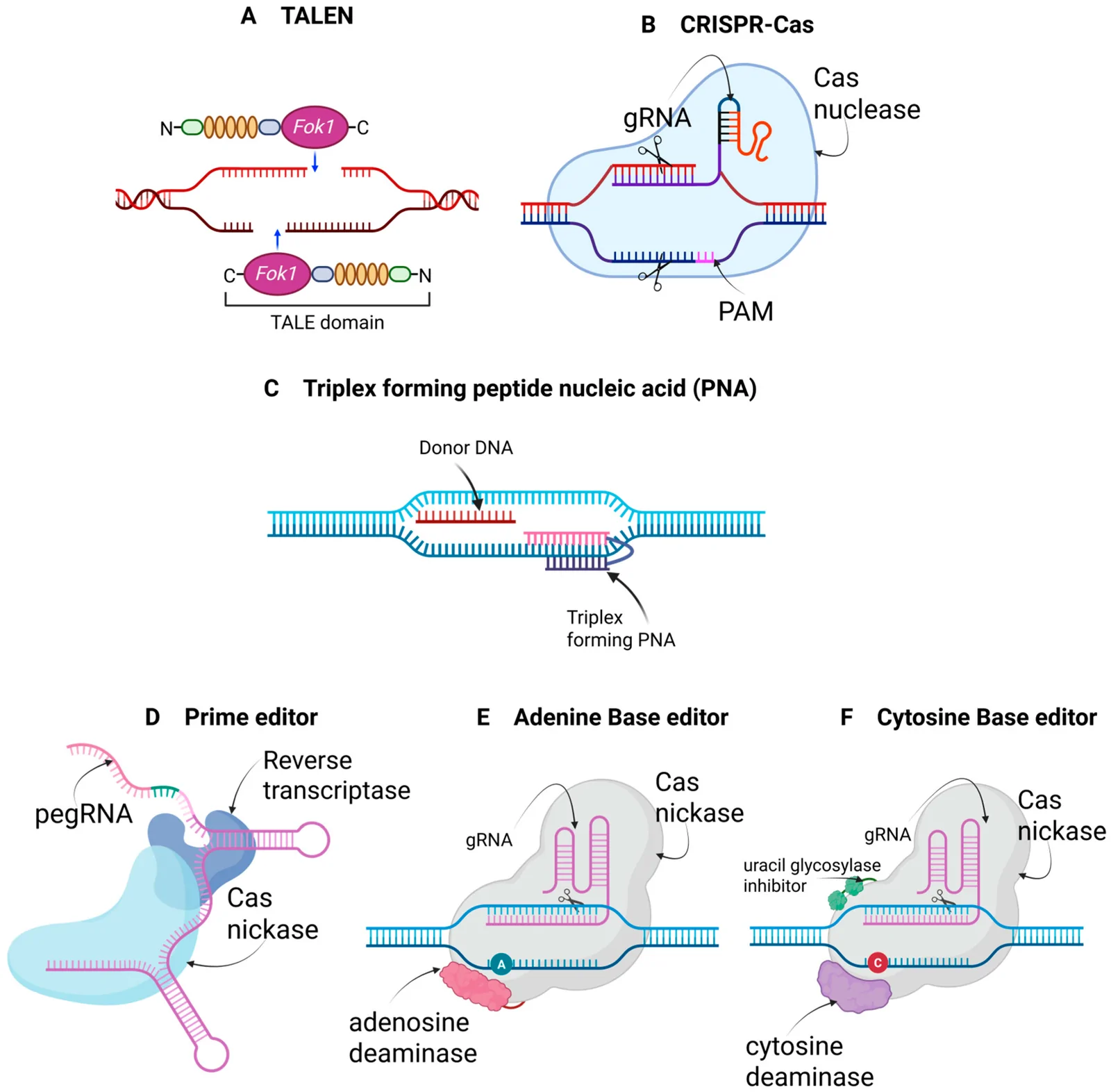
Schematic of gene-editing tools that have been generated to target and manipulate DNA sequences. (**A**) Transcription activator-like effector nucleases; (**B**) CRISPR-Cas; (**C**) Prime editor; (**D**) Triplex forming PNA; (**E**) Adenine base editor; and (**F**) Cytosine base editor. Created in BioRender. Oglesby, I. (2026) https://BioRender.com/tbbnp08, accessed on 21 September 2026.

Recent decades have seen the development and advancement of multiple gene-editing tools, enabling precise manipulation of the genome with increasing efficiency, specificity, and versatility. Targeted editing utilises nucleases guided by proteins or nucleic acid sequences such as Transcription Activator-Like Effector Nucleases (TALEN), CRISPR, Base/Prime editors and triplex-forming PNAs (Figure 1).

Early gene editing technology such as TALENs consist of linked proteins bound to a Fok-1 enzyme and operate as heterodimers. The major limitations associated with TALENs include their large size, making delivery more challenging, and complex TALE protein engineering [17]. The delivery challenges associated with the large size of TALENs can in some cases be addressed in vivo and in vitro, through the use of mRNA [18,19]. TALENs’ main advantage is their ability to target highly repetitive sequences, such as heterochromatin, which can be dysregulated in smoking-related lung diseases like COPD [20]. TALENs have been utilised to generate several lung-based models by inserting reporters and creating isogenic pairs [21,22,23].

To overcome difficulties posed by protein-DNA targeting, CRISPR systems use guide RNAs (gRNA) to direct CRISPR-associated protein (Cas) nucleases to complementary DNA sequences adjacent to a protospacer adjacent motif (PAM), enabling targeted DNA cleavage or modulation [17]. Compared to TALENs, CRISPR targeting is simpler, as it is controlled by redesigning the gRNA [24]. Cas9 is the most widely used nuclease and generates blunt double-strand breaks; other options, including Cas12a, are becoming popular. Cas9 has been utilised for well-validated editing across lung epithelial cells and immune cells [25,26,27]. Cas12a creates staggered DNA ends, and like Cas9, robustly edits immune cells [28,29]. Hammad et al. recently described CRISPR/Cas12a-Ultra, an engineered Cas12a with increased efficiencies, in hard-to-transfect cell types, including iPSCs [29]. Immunogenicity of Cas proteins have emerged as a possible roadblock to clinical use, with potential activation of the innate and adaptive immune system. Several strategies to address this have emerged, including immunosuppression, epitope masking on the Cas surface or the use of non-pathogenic bacterial Cas proteins [30].

A major recent advance has been the development of compact nucleases designed to overcome the packaging constraints of adeno-associated viruses (AAVs). Among these, Cas12f stands out due to its exceptionally small size, enabling packaging within a single AAV vector [31]. Engineering efforts that enhanced DNA binding and catalytic efficiency by Guan et al. have further improved the activity of the Al3Cas12f RKK variant, increasing editing efficiency from less than 10% to approximately 80% [31]. These compact editors are particularly promising for lung-directed therapies, where single-AAV delivery, efficient tissue penetration and minimal immunogenicity are critical.

Base- and prime-editors are newer tools that utilise a Cas nickase or non-cleaving Cas (dCas). Cas nickases do not create a DSB, as one nuclease domain is inactivated, reducing the risk of chromosomal rearrangements and p53 activation [32]. There are two classes of base-editing, adenine base-editing (ABE), and cytosine base-editing (CBE) [33]. CBEs alter the target nucleotide from Cytosine (C) and Guanine (G) to Thymine (T) and Adenine (A) via a cytosine deaminase converting a C to an uracil base that DNA polymerase reads as T. ABEs utilise adenosine deaminase to facilitate A and T to G and C changes, where A is converted to inosine, which is read as a G by DNA polymerase [33]. Adenine base editing has been shown to correct the Glu342Lys AATD1 variant in patient-derived iPSCs [34].

Prime-editors combine a Cas nickase, a reverse transcriptase and a prime-editing guide RNA (pegRNA) that encodes the desired edit. Therefore, it is a powerful tool for correcting pathogenic variants in lung disease models where homologous repair is impaired, as no repair template is required, but may have lower efficiencies than other less complex systems [35]. Fiumara et al. compared traditional Cas targeting, CBE, ABE and PE approaches to edit hematopoietic stem cells and found that base and prime editors are still capable of creating double strand breaks, providing a risk for large deletions and translocations and that CBEs were the most likely nickase system for this to occur in. Beyond DNA-breaks all systems caused cellular stress that affects editing efficiency and clonogenic capacity [36]. In some cases, base-editors can have off-target RNA editing as a result of their deaminase acting on RNA substrates in both coding and non-coding regions. Modifications to the deaminases and the use of transient delivery approaches, such as RNPs or mRNA can help to reduce unintended RNA editing [37].

PNAs address many of the remaining concerns with CRISPR as they have higher binding stability, lower off-target effects and greater specificity [38]. PNAs are synthetic DNA analogues that bind complementary nucleic acids more tightly than DNA–DNA or RNA–RNA duplexes and do not tolerate base mismatches [38,39]. Triplex-forming PNAs can be used in DNA editing by causing a helical distortion that activates DNA repair mechanisms. This would be highly valuable for applications requiring ultra-high specificity, such as editing airway or alveolar progenitor cells, where off-target events could have long-term consequences resulting in the recombination of a DNA donor near the PNA binding site [39]. The use of PNAs for gene editing is still in its infancy, with further work needed to understand mechanisms and better define design guidelines.

CRISPR-based transcriptional modulators, such as CRISPR activation (CRISPRa) and CRISPR interference (CRISPRi) were first described by Qi and colleagues in 2013 [40,41]. By using a dCas9 and co-expression with a sgRNA, specific silencing (CRISPRi) or activation (CRISPRa) of the gene of interest can be achieved, with reduced of off-target effects and toxicity as a key advantage of these tools [42]. Multiple genes can be reversibly suppressed simultaneously. Since its development, these techniques have been used for the investigation of different diseases. Stuart and colleagues investigated lung disease-associated loci for CF, COPD and IPF that were identified by genome-wide association studies (GWAS) using CRISPRi [43]. They showed that CRISPRi offers a method to analyse the function of non-coding regions and their effect on gene-regulation. This approach has since been used by other groups to investigate the impact of genomic regions in lung diseases [44,45]. Furthermore, Liu et al., used CRISPRa to investigate the role of CEBPA in fibroblasts [46]. They found that CEBPA gene expression is significantly downregulated in IPF-derived fibroblasts. Together, these studies demonstrate the potential of modified CRISPR variants for the investigation of specific loci and their impact on diseases as well as future therapies, due to their reversibility and low toxicity.

Together, these gene-editing tools provide a versatile platform to model chronic respiratory diseases and hold considerable promise for future therapeutic applications. CRISPR and CRISPR-derived systems, including CRISPRa, CRISPRi, base editors, and prime editors, offer distinct advantages for studying and correcting disease-associated genetic alterations, owing to their ease of targeting, flexibility, and varying editing efficiencies. In particular, base and prime editors enable precise genetic modification without the need for double-strand DNA breaks or exogenous donor templates, reducing the risk of unwanted genomic alterations. PNAs are comparatively new but represent an attractive alternative due to their high specificity and lack of reliance on nuclease activity, potentially reducing the risk of off-target effects and genomic instability. It is important to recognise that structural aberrations such as indels, large deletions, chromosomal truncations, and translocations remain potential risks, which are discussed in Aussel et al. [47]. Beyond this, editing in human embryos and germline cells remains an ethical concern. This issue is reviewed thoroughly in Reza Sadeghi’s editorial article [48]. The changes in clonogenic capacity, increased cellular stress, and genomic instability discussed above may potentially contribute to tumorigenic processes. This is particularly important to consider when editing long-lived resident progenitor cells in the airway and lungs, where genetic alterations could affect proliferative capacity, genomic integrity, and homeostasis.

Successful translation of these technologies to patients will depend not only on selecting the most appropriate editing tool for a given disease-causing mutation, but also on pairing these tools with efficient and safe delivery systems. Furthermore, rigorous validation in physiologically relevant human models will be essential to assess efficacy, safety, and long-term outcomes before clinical implementation.

## 3. Barriers to Pulmonary Delivery

Efficient and safe delivery of gene-editing tools to the lung remains one of the most significant challenges in developing therapies for the lung. The lungs complex anatomy, dynamic physiology and multilayered defence systems create a series of physical, biochemical, and immunological barriers that limit access of viral and non-viral vectors to target cells. Understanding these barriers is essential for designing delivery systems capable of achieving therapeutically meaningful editing in airway and alveolar compartments.

Permanently exposed to the external environment, the lung is structurally designed to protect the airway cells against inhaled particles, allergens, and pathogens. Branching airways within the lung allow air to reach millions of alveoli thereby facilitating gas exchange. However, these branches create a complex structure that influences nanoparticle delivery and distribution. Intratracheal instillation of bleomycin in mice is a widely used model to study IPF, but the heterogeneous distribution of fibrotic lesions between lungs remains a major drawback of the model [49,50]. Development of new drug formulations, such as aerosolization of bleomycin via the intratracheal route has improved this limitation in rats [51]. Aerosol particle size is key in dictating its successful delivery to its targeted site in the lung. Large particles (>5 μm) are usually retained in the bronchial regions by impaction, whereas particles of 1–5 μm tend to sediment under gravitational force in the bronchioles and alveoli where the airflow is low [52]. On the other hand, smaller particles (<0.5 μm) have very little deposition in the alveoli by diffusion as they are mostly exhaled due to their small size [52].

The airway mucus layer, composed primarily of MUC5AC and MUC5B mucins and the underlying periciliary liquid (PCL), form the first major barrier to gene delivery. The airway surface layer immobilises trapped particles and provides lubrication for ciliary beating, enabling movement of foreign materials towards the pharynx by coughing or swallowing. This mechanism known as mucociliary clearance, also restricts diffusion of nanoparticles and viral vectors, posing a major challenge [53]. In CF, a mutation in the gene coding for the cystic fibrosis transmembrane conductance regulator (CFTR) protein leads to an imbalance between sodium ion absorption and chloride ion secretion, altering mucus clearance [54]. This significantly obstructs nanoparticles from penetrating the thickened mucus and moving to the epithelial cells for drug uptake [55]. COPD is also characterised by overproduction of mucus and limited airflow rate, thereby compromising pulmonary deposition and inhalable aerosol distribution [56].

Furthermore, beneath the mucus and periciliary layer, tight junctions maintain epithelial barrier integrity and apical–basolateral polarity [57], which is essential for the normal physiological function of epithelial cells. This structure limits access to many receptors required for the internalisation of viral vectors, which are located on the basolateral surface [58], such as AAVs. Disruption of the tight junction integrity and apical–basal polarity by the knockout of Crumbs 3 has been demonstrated to influence the localisation of glycans to the cell surface, enhancing AAV attachment and transduction [59].

Pulmonary surfactant, composed of phospholipids and proteins, forms a scaffold that regulates surface tension and prevents the collapse of alveoli [60]. However, when in contact with nanoparticles, it results in aggregation or swelling, impairing uptake, and creating a solid defence system against gene delivery to epithelial cells [61]. Interestingly, the interaction of nanoparticles with the pulmonary surfactant system can be a precursor to the immunological barrier to gene therapy. Approximately 10% of surfactant consists of protein. Of the four surfactant proteins, the immunomodulatory roles of SP-A and SP-D have been well studied [62]. As members of the collectin family of proteins, SP-A and SP-D can bind inhaled particles, including nanoparticles and increase uptake by the immune cells [62].

In addition to structural defences, the lung contains a dense network of immune cells, such as alveolar macrophages, dendritic cells and interstitial macrophages that can all significantly hinder the delivery of gene-editing therapies. Alveolar macrophages actively inhibit the uptake of delivered vectors via phagocytosis, while also inducing antiviral responses that suppress transgene expression [60]. These immune barriers are amplified in diseased lungs, where inflammation, fibrosis or infection heighten macrophage activation and accelerate vector clearance.

Ribonuclease also contributes to inefficient gene therapy delivery. Ubiquitous DNases and RNases in the airway and alveolar fluid regulate nucleic acid degradation, resulting in reduced stability of mRNA-LNPs and plasmid-based delivery vectors [63]. In IPF, excessive synthesis and deposition of extracellular matrix (ECM) components form a physical barrier to vector diffusion and penetration of mRNA-mediated therapies [64].

In addition to these structural and immunological barriers, emerging evidence highlights the importance of cell-intrinsic biological barriers that influence the efficiency of gene-editing delivery. Recent work has shown that the metabolic state of the target cell is a key determinant of LNP uptake and mRNA expression. Under physiologically relevant, plasma-like conditions, LNP efficiency is significantly reduced due to suppression of amino acid pathways. Supplementation with methionine, arginine, and serine (AAS) markedly enhanced LNP uptake and mRNA expression. Co-administration of AAS with LNPs resulted in a 5–20-fold increase in mRNA expression across multiple cell types, delivery routes, and formulations, both in vitro and in vivo. This highlights metabolic modulation as a promising strategy to overcome current delivery inefficiencies [65], particularly in diseased lung environments where epithelial metabolism is altered by inflammation, fibrosis, hypoxia and oxidative stress.

Together, the lungs’ complex structure and multi-layered defence system, including mucus, surfactant, epithelial polarity, immune surveillance, and dynamic clearance give rise to formidable obstacles to in vivo gene editing. Overcoming these barriers requires rational vector engineering, surface modification strategies, aerosol-compatible formulations, and human-relevant models to evaluate delivery performance. Addressing these challenges will lead to prolonged therapeutic efficacy in the lungs and safer targeted pulmonary therapy.

## 4. Platforms for Delivering Gene Therapies to the Lungs

Drug particle delivery to the lung is an ongoing clinical challenge and an emerging research area in respiratory gene therapy. Effective in vivo gene editing in the lung requires delivery systems capable of navigating the anatomical, biochemical, and immunological barriers outlined above. In addition, off-target effects and the potential for cumulative or unanticipated toxicity, especially with repeated dosing, raises further safety issues. In vivo, delivery platforms of gene-editing tools to the lung can be broadly classified into viral and non-viral vectors, as summarised in Table 1. Each offers distinct advantages and limitations for pulmonary applications.

Among viral vectors, adeno-associated virus vectors (AAVs) are the most clinically advanced platform due to their favourable safety profile and efficient transduction of mammalian tissues [66]. They are non-enveloped viruses with a 4.7kb ssDNA genome. Due to their small capacity, a dual AAV system has been developed to double the capacity. AAVs have been utilised in in vivo studies as they are reported to have mild immunogenicity [67]. AAVs can trigger both innate and adaptive immune responses. Serval tactics to ameliorate these responses are used, for example the deletion of the CpG motif to evade toll-like receptor activation or capsid engineering. These strategies are reviewed in Arjomandnejad et al. [68]. As there are naturally occurring AAVs, neutralising antibodies are found in the healthy population and can contribute to reduced efficiency of common AAVs [68,69]. Distinct AAV variants or serotypes can preferentially transduce distinct cell types; those employed in the lung are AAV1, AAV4, AAV5, AAV6, and AAV9 [70]. Dual AAV systems are often used to deliver the pyogenes Cas9 (spCas9) gene in one AAV, as it is ~4.2kb, requiring the majority of a single AAV’s capacity and a second AAV is therefore used to deliver the gRNA and homology-directed repair template strand if applicable [71]. Dual recombinant AAVs have been developed to deliver base editors as multiple components [67].

Helper-dependent (HD) adenoviruses do not contain any viral coding sequences, giving them a large carrying capacity of ~36 kb [72]. The lack of viral genes results in lower immunogenicity. Given HD cannot replicate, they must be co-transduced with a second virus when expanding. This virus may remain as a contaminant. Therefore strategies to prevent this are needed before use in vivo [72]. Studies by Cao et al. showed that HD-AdVs can efficiently deliver transgenes to airway epithelial cells in mouse, rabbit, and pig lungs, as well as to human primary epithelial cells, with a further study showing comparable results in mouse and pig airway basal cells [73], underscoring their suitability for respiratory gene-delivery applications.

Lentiviruses (LV) are retroviruses with a lipid envelope and an ~10.7 kb ssRNA genome with the ability to stably integrate into their hosts genome. LVs use their glycoprotein envelope to bind and enter cells, with the ability to transduce non-replicating and terminally differentiated cells, such as alveolar type 1 cells [74]. The immunogenicity of LVs and immunomodulatory approaches to mediate immune responses are covered in Annoni et al. [75]. It should be noted that LVs are not a suitable vector when working with TALENs, as repeats in the TALENs are deleted during packaging, leading to potential mutagenesis [76]. Integrase-deficient LV (IDLV) have been optimised to reduce integration, lowering toxicity, and minimising off-target effects [67].

Non-viral systems have emerged as an attractive alternative due to their capacity for repeat dosing and transient gene-editing expression. Lipid Nanoparticles (LNPs) are non-viral vesicles that are bound by one or more lipid-bilayers and are extensively reviewed by Elrufaie et al. [77]. Cationic LNPs are appropriate delivery vehicles for TALENs and CRISPR. They have a larger carrying capacity than most viral vectors, allowing them to carry Cas9 proteins. Cationic LNPs can also carry plasmids encoding for TALENs and CRISPR components [45]. Ribonucleoproteins (RNPs) may also be used to deliver Cas9 and ssDNA templates [78]. RNPs are expected to have lower toxicity and immunogenicity with lower toll-like receptor (TLR) activation than mRNA delivery. It may also provide lower off-target effects as the Cas protein has a short half-life when administered [78]. RNPs face challenges with LNP encapsulation, including a lack of sufficient the negative charge required for encapsulation and the frequency of protein denaturation that occurs with the organic solvents used in LNP formulation [78].

More recently, Selective Organ Targeting Lipid Nanoparticles (SORT LNPs) have been utilised to deliver CRISPR to lung basal cells [79]. Wei et al. used a SORT LNP to intravenously deliver CRISPR/Cas9 components to initiate a DSB and homology-directed repair (HDR) in the CFTR gene. They found that this system could transfect ~60% of the lung basal cell population after systemic IV administration in mice.

Another non-viral delivery option is 5–30 amino-acid long cell-penetrating peptides (CPPs) that readily cross the cell membrane to facilitate direct membrane translocation of gene-editing cargos, particularly when conjugated to RNP complexes, although their uptake efficiency and in vivo stability remain variable [80]. Nebulised nanoparticles processed using the four-step LOOP method have a high helper lipid ratio and buffer that improve mRNA expression in the lung, with the ability to better withstand the sheer force damage that occurs during nebulisation [81].

Overall, viral vectors such as AAVs, HD-adenoviruses, and lentiviruses offer high efficiency and, in some cases, large cargo capacity, but are limited by immune recognition, safety concerns, and packaging constraints. While both AAVs and LVs have attractive qualities, AAVs non-integrating nature and stable transgene expression make them the current forerunner [82]. Non-viral systems, particularly lipid nanoparticles, CPPs, and nebulised formulations, provide safer and more flexible delivery with repeat dosing potential, though they currently suffer from lower targeting efficiency and variable in vivo performance. The deposition of gene-editing tools along access routes, such as the trachea, small airways or in the circulatory system, and biodistribution outside the key cell type remains a concern. This highlights the need for lung-specific delivery platforms and a lack of biodistribution data in humans [83]. While significant progress has been made in both viral and non-viral delivery of tools for pulmonary gene editing, there is not one system that achieves the balance of efficiency, safety, and lung-specific targeting, leaving a need for continued innovation in delivery design.

**Table 1:** Comparative table of platforms for gene-editing tool delivery.

	Viral			Non-Viral	
	Adeno-Associated Virus Vector [67,68,84]	Helper-Dependent Adenovirus Vectors [85,86,87]	Lentivirus [74,75,84]	Lipid Nanoparticle [88,89,90]	Direct Delivery and Cell Penetrating Peptides [91]
**Structure**	Non-enveloped viruses with single-stranded DNA	Non-enveloped virus with double stranded DNA, devoid of viral coding sequences	Enveloped virus with single-stranded RNA	Vesicles bound by one or more lipid-bilayer	Short peptides (5–30 amino acids) bound to cargo
**Immune** **response**	Potential innate and adaptive immune response	Potential innate response but attenuated adaptive response	Potential innate and adaptive immune response	Various production methods have toxicity and mutagenesis concerns [30]	Not applicable in vivo
**Maximum viral titre infection units/mL**	1 × 10^9^	1 × 10^13^	1 × 10^9^	-	-
**Integrating**	Non-integrating	Non-integrating	Integrating	-	-
**Cell cycle** **targeting**	Both dividing and non-dividing cells	Both dividing and non-dividing cells	Both dividing and non-dividing cells	Both dividing and non-dividing cells	

## 5. Gene-Editing in Human Preclinical Models

Progress in lung-directed gene editing increasingly depends on preclinical models that accurately reproduce human airway and alveolar biology. Traditional murine models, while indispensable for in vivo delivery, do not fully recapitulate human epithelial composition, immunogenicity, surfactant biology, or telomere-associated disease mechanisms. Therefore, there has been a shift toward human-relevant platforms that enable the precise evaluation of editing efficiency, specificity, and safety prior to clinical translation. However, no single model fully recapitulates the complexity of the human lung, with each having biological and technical constraints that must be considered (as summarised in Table 2).

ALI cultures represent one of the most widely used primary human airway models. In ALI systems, primary airway epithelial cells are cultured on a collagen matrix in transwell inserts. The apical surface of the cells is exposed to air while the basolateral surface has access to the growth media. This configuration induces key morphological, molecular, and functional features of the native airway epithelium, including pseudostratification, tight-junction formation, ciliation, mucus secretion and mucociliary transport [92]. These features make ALI cultures particularly valuable for assessing vector tropism, epithelial barrier penetration, and apical delivery strategies. Furthermore, studies have shown a close resemblance of ALI cultures and primary cultures at the transcription level [93,94], highlighting the physiological relevance of this platform.

ALI studies have also been instrumental to uncovering key mechanistic barriers for pulmonary gene delivery. In the context of CF pharmacotherapies, the ALI model has proved its importance by highlighting the need for agents that are capable of permeabilizing the apical cell border. In vivo studies in mice and explanted human lung tissue demonstrate that effective adenovirus vector transduction is only possible after epithelial wounding or exposure to medium-chain fatty acid sodium caprate (C10) [95,96]. Furthermore, combining advances in aerosol exposure systems with ALI have enabled controlled testing of nanoparticles, complex mixtures, dry powders, and liquid or gas aerosols for inhalation under physiologically relevant conditions [97,98].

These models were mainly tested using cell lines such as A549 or THP-1. Combining these models with primary human cells would enhance their translatability. However, major limitations to these models include limited access to primary human cells and patient-to-patient variability. Additionally, primary cells exhibit finite proliferative capacity, before exhibiting phenotypic abnormalities caused by epigenetic modifications [99]. In addition, the absence of other cell types, such as immune, stromal, and vascular components limits their ability to model inflammatory responses and systemic delivery, potentially leading to overestimation of editing efficiency and underrepresentation of immunogenicity [100,101].

To address some of these limitations, iPSC-derived lung models have emerged as reproducible and genetically defined complementary platforms. iPSCs can be generated efficiently from somatic cells, such as peripheral blood mononuclear cells, enabling patient-specific disease modelling without the ethical constraints associated with embryonic stem cells. Through directed differentiation, iPSCs can generate alveolar epithelial type I (AT1) and II (AT2) cells, airway basal cells, and macrophages that capture key transcriptional and functional features of their in vivo counterparts [23,102,103,104,105].

A major strength of iPSC-based systems lies in their compatibility with gene-editing technologies to generate isogenic disease models. In CF, correction of *CFTR* mutations in patient-derived iPSCs has enabled direct comparison between mutant and repaired genotypes, with functional rescue demonstrated using assays such as forskolin-induced swelling in spheroids and electrophysiological measurements in ALI cultures derived from iPSC airway basal cells [102,104,106,107,108]. These approaches are particularly valuable for studying rare variants that are not addressed by existing therapies [109]. Beyond CF, iPSC models have been applied to a range of monogenic lung diseases. In AATD, base editing has been used to correct the *SERPINA1* Z mutation in patient-derived iPSCs with minimal detectable off-target effects, highlighting the potential of precise nucleotide editing strategies [34]. Similarly, CRISPR- and TALEN-based approaches have been used to model and correct surfactant protein mutations associated with interstitial lung disease, revealing defects in protein processing and trafficking [110]. Gene-edited pluripotent stem cell models have also been used to study fibrotic disorders such as Hermansky–Pudlak syndrome, where engineered mutations recapitulate transcriptional signatures observed in patient-derived pulmonary fibrosis [111,112].

These models have advanced the mechanistic understanding of these diseases and have enabled controlled evaluation of rare variant diseases. Despite these advances, important limitations remain. iPSC-derived lung cells frequently exhibit developmental immaturity, with transcriptional and functional profiles resembling foetal rather than adult tissue. Differentiation efficiency can vary significantly between cell lines and protocols, introducing experimental variability and limiting reproducibility [113]. These factors may influence gene-editing outcomes, including DNA repair pathway activity and vector uptake. As a result, editing efficiencies observed in iPSC-derived systems may not fully translate to adult human lung tissue.

Three-dimensional (3D) lung organoids extend these approaches by incorporating multicellular architecture and enabling cell–cell interactions not captured in two-dimensional systems. Organoids are self-organising structures derived from stem cells that recapitulate aspects of tissue architecture, whereas spheroids represent simpler aggregates typically composed of a single cell type. Three-dimensional lung organoids can be generated from iPSCs, embryonic stem cells, or adult stem cells [114,115,116]. Rather than generating one specific cell type of the lung, lung organoids are composed of multiple cell types, supporting more complex behaviours. Gene editing has been successfully applied in these organoid systems, including *NKX2-1* activation, *ACTB* locus transgene integration, and *TP53* knockout in a human-pluripotent-stem-cell-derived alveolar organoid system [117]. However, most organoid systems lack key components of the in vivo environment, including vascularisation, mechanical forces associated with breathing, and fully functional immune compartments [113]. Structural heterogeneity between organoids and limited access to luminal surfaces can also complicate delivery of gene-editing reagents. Consequently, organoid systems are well suited for studying cell-intrinsic responses to gene editing but are less predictive of in vivo delivery dynamics.

Lung-on-a-chip models aim to address some of these limitations and extend physiological relevance by incorporating simulated dynamic breathing, mechanical stretch, co-culture systems and microfluidics, to model dynamic aspects of lung physiology, including airflow, vascular perfusion, barrier function and immune–epithelial interactions [118,119,120,121]. These systems provide unique opportunities to study how physical forces and flow conditions influence gene delivery and editing efficiency. Using gene-edited cells in lung-on-a-chip models, Mattoscio and colleagues investigated the multifunctional role of *CFTR* [122], demonstrating the utility of these platforms for mechanistic studies. Nevertheless, lung-on-a-chip platforms remain technically complex and relatively low throughput, which limits their application for large-scale screening [123,124]. In addition, variability between device designs and a lack of standardisation complicate cross-study comparisons.

Finally, at the highest level of physiological relevance are ex vivo human lung models, such as precision cut lung slices (PCLS) and ex vivo lung perfusion (EVLP) models. PCLS are thin slices of lung tissue that maintain the native architecture and cell-to-cell interactions of the lung tissue, enabling short-term assessment of gene editing in human lung tissue. However, their lack of circulation, tissue viability over time, lack of standardisation protocols and significant donor-to-donor variability pose major challenges [125]. Diffusion barriers within tissue slices may also restrict uniform delivery of gene-editing reagents. EVLP models allow whole-lung perfusion and manipulation outside of the body, creating a unique opportunity to evaluate delivery, editing efficiency and tissue responses at organ scale. Recent work by Mesaki et al., demonstrated the feasibility of a genetic engineering strategy to modulate immune responses in lung grafts and successfully carried out genome editing in human lungs ex vivo through organ perfusion [126]. Genome editing was assessed for an extended period of 14 days in live lung tissue using PCLS [126]. Additionally, Mesaki and colleagues tested the potential of CRISPR-mediated transcriptional upregulation of cytokines in rat lungs and successfully transplanted the CRISPR-edited lungs into recipient rats demonstrating the potential of ex vivo genome-editing to improve transplant outcomes [126]. These findings highlight the promising potential of utilising ex vivo human organs as a platform for preclinical evaluation of gene-targeted therapies, bridging the gap between reductionist in vitro systems and in vivo studies. However, despite these advantages, EVLP is constrained by limited organ availability, short experimental windows, high cost, and logistical complexity. Furthermore, both PCLS and EVLP lack full systemic immune integration, which remains a critical determinant of gene-editing safety and durability in vivo.

Together, these platforms provide a continuum of human relevant systems, ranging from reductionist to organ level complexity models, that enable rigorous evaluation of gene-editing efficiency, specificity, delivery performance, and tissue-level toxicity. Rather than viewing any single model as definitive, their complementary strengths and limitations highlight the need for integrated, multi-platform evaluation strategies. Iterative testing across these systems is essential to accurately assess gene-editing efficiency, delivery performance, specificity, and safety, so that the development of lung-directed gene-editing therapeutics can be accelerated, helping to overcome key barriers in translational research.

## 6. Translating In Vitro Studies into Future Therapeutics

Successful translation of respiratory gene-editing therapies requires integration of the disease mechanism, target cell population, delivery strategy, and required durability of the genetic correction. While preclinical models enable evaluation of editing efficiency, specificity, and toxicity, their translational relevance ultimately depends on how accurately they reflect the cells that must be modified in vivo to achieve durable therapeutic benefit.

A key principle emerging from respiratory gene-editing studies is that long-term therapeutic efficacy generally requires targeting tissue-resident stem and progenitor cell populations. In the airway epithelium, basal stem cells function as key regenerative units capable of sustaining epithelial renewal, making them a primary target for durable correction in diseases such as CF [127,128]. Similarly, in the distal lung, AT2 cells, the alveolar epithelial progenitors, are key therapeutic targets in diseases such as PF, where alveolar integrity and repair are disrupted.

The importance of aligning disease biology with target cell type is further illustrated by systemic respiratory genetic disorders such as AATD, where hepatocytes rather than airway epithelial cells represent the primary therapeutic target due to their role in systemic protein production. In such cases, liver-directed in vivo mRNA-encoded adenine base-editing using LNP delivery strategies is being actively explored highlighting how target organ selection fundamentally determines delivery route and therapeutic design. In contrast, CF and other airway diseases require delivery to basal stem cells, where delivery barriers such as mucus accumulation, epithelial tight junctions, and limited vector penetration significantly constrain the efficiency of in vivo editing approaches and strongly influence the choice between viral, non-viral, or ex vivo strategies, reviewed thoroughly in reference [7].

Overall, successful gene-editing strategies for respiratory disease require alignment of the disease mechanism, target cell type, editing modality, and delivery route. Preclinical models therefore serve not only as tools for assessing technical performance but also for defining the most appropriate cellular targets for durable therapeutic correction.

## 7. Emerging Gene-Editing Therapeutics

**Figure 2:**
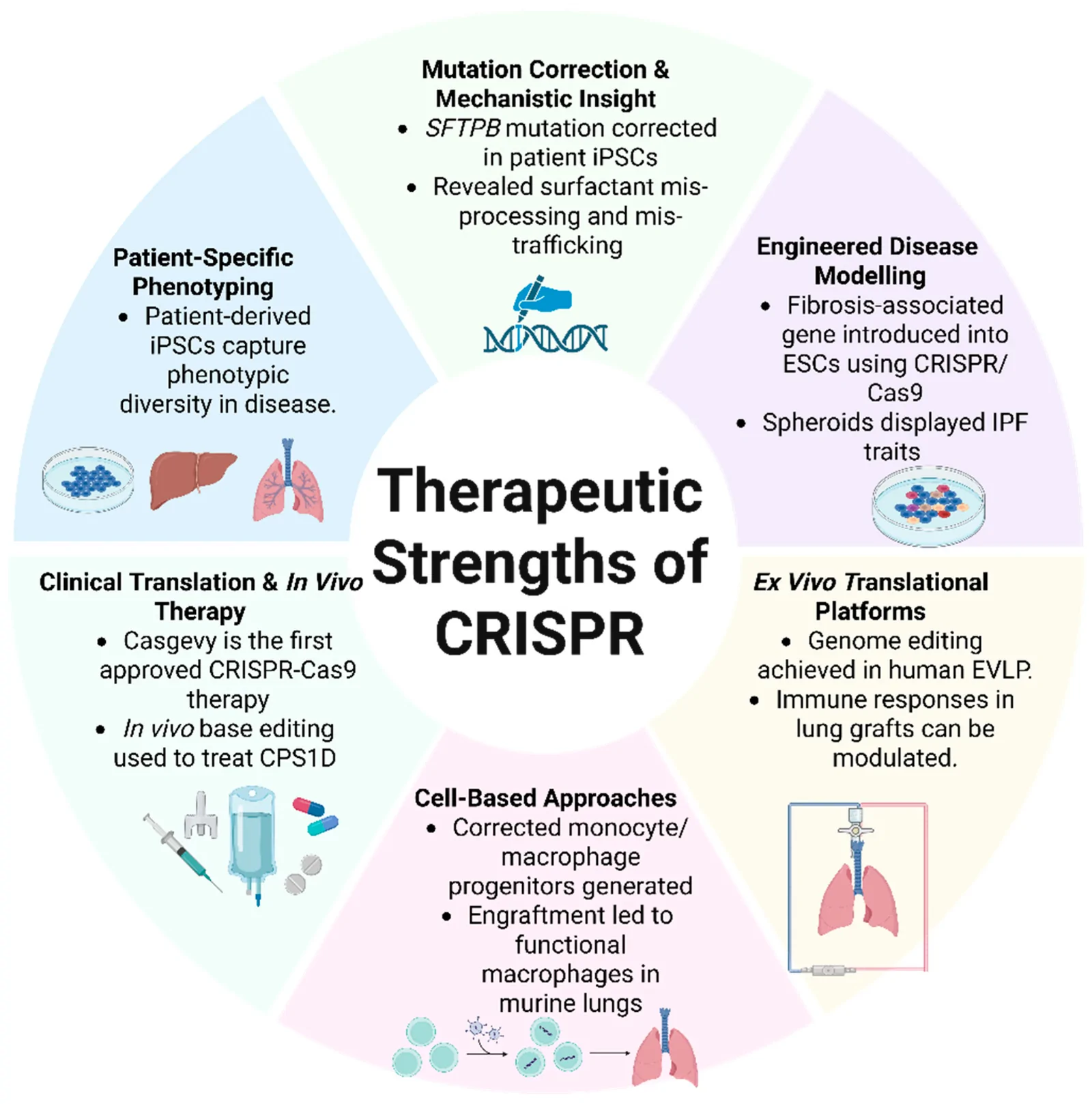
Therapeutic strengths of CRISPR in patient-specific phenotyping, mutation correction and mechanistic insight, engineered disease modelling, ex-vivo translational platforms, cell-based therapeutic approaches, clinical translation, and in vivo therapy. Created in BioRender. Oglesby, I. (2026) https://BioRender.com/zwmgqg9, accessed on 21 September 2026.

Emerging gene-editing therapeutics are beginning to demonstrate clinical feasibility across a spectrum of rare and complex diseases, providing a framework for future applications in the lung. Although pulmonary gene-editing therapies remain limited, recent advances in progenitor and in vivo editing and regenerative cell transplantation highlight the expanding therapeutic landscape. The therapeutic potential and strengths of CRISPR based methods are outlined in Figure 2.

One promising strategy involves ex vivo correction of patient-derived progenitor cells followed by autologous transplantation. Kuhn et al. demonstrated functional correction of human iPSC-derived macrophages using TALEN targeting [21]. Using this technique, they were able to generate functionally corrected monocytes and macrophages, capable of restoring defective function. This work builds on previous studies in hereditary alveolar proteinosis (herPAP), where transplantation of alveolar macrophage progenitors into murine lungs resulted in successful in vivo lung-specific engraftment between 6 weeks and 9 months post-transplantation [129,130]. Transplantation into humanised mouse models also resulted in a significant and long-lasting improvement of the disease phenotype, showing the potential of gene-corrected progenitors for lung disorders.

In parallel, rapid progress in in vivo gene-editing is beginning to demonstrate clinical feasibility. A landmark example was recently reported in the rare genetic disease carbamoyl-phosphate synthetase 1 deficiency (CPS1D), a metabolic disorder that results in an accumulation of ammonia that leads to damage in the lungs and brain [131]. After two administrations of the base-editing therapy via LNPs at the age of 7 and 8 months, a male neonate improved his intake of dietary protein and required less nitrogen-scavenger medication. Although the authors highlight the need for a longer follow-up to assess the safety and efficacy of their treatment, an increased weight and a stable neurologic status was seen at the end of the 7-week follow-up period. This study highlights the feasibility of gene-editing as a therapy and lays the path for future targeting in the lung and beyond.

Building on this study, early clinical and preclinical trials are now targeting diseases with pulmonary relevance, including conditions with systemic manifestations (Table 3). AATD is a genetic disorder that causes low levels of circulating AAT protein, this leads to liver and lung damage. Recent developments in in vivo gene-editing for AATD underscore the rapid progress in clinically relevant editing technologies. BEAM-302 (NCT06389877) is a Phase1/2, study, evaluating an mRNA-encoded adenine base-editor targeting the liver delivered via LNPs for adults with PiZZ AATD-associated lung and/or liver disease [7]. Although this study targeted the liver, it represents one of the first clinical applications of in vivo base-editing for a genetic lung-associated disorder, providing a critical precedent for future pulmonary gene-editing trials. Complementing this, CRISPR Therapeutics’ CTX460, developed using the SyNTase^TM^ editing platform demonstrated highly efficient in vivo correction of the *SERPINA1* E342K (PiZZ) mutation in preclinical AATD models, where a single LNP-delivered dose achieved >90% mRNA correction, and a >5-fold increase in total AAT [132]. Together, these studies illustrate the accelerating translation of in vivo gene-editing toward diseases with pulmonary manifestations.

Beyond direct therapeutic use, CRISPR technologies have become indispensable tools for target discovery and mechanistic interrogation. CRISPR screening, in pooled or arrayed formats is a high-throughput functional genomics technique to investigate the function of specific genes [133]. Pooled screens use Cas9-expressing cells in bulk, and rely on sequencing as a read-out, whilst arrayed screens physically separate between target genes and therefore other read-outs like imaging, proteomics and metabolomics are sufficient. This makes pooled screens more suitable for discovery, and arrayed screens for validation and follow-up investigation.

Complementary advances in bioinformatic tools and databases have accelerated single guide RNA design, and off-target detection techniques, e.g., CHOPCHOP and CRISPRscan [134,135,136], as highlighted in recent reviews [137,138,139]. Emerging platforms such as Gene Knockout Interference (GenKI) can be used to support CRISPR techniques by in silico knockdown analysis [140]. This tool can create virtual knockouts to predict gene function in single-cell RNA sequencing datasets without the need to include knockout samples in the sequencing. Together, these studies highlight the development of wet lab and computational innovations that go hand in hand to enhance the efficiency, scalability, and interpretability of CRISPR-based discovery pipelines.

Recent studies have also exploited the use of iPSC-derived pulmonary cells as regenerative therapies for lung disease in mice [141,142,143]. These reports detail the reconstitution of both the proximal and distal lung epithelium following transplantation of pluripotent stem cell-derived cells. Engraftment cells exhibited self-renewal and multi-lineage airway and alveolar epithelial differentiation in multiple mouse models for up to 2 years after transplantation. This shows that engineered pulmonary cells may serve as a platform for future gene-correct, cell-based therapies.

## 8. Conclusions and Future Directions

In recent decades, gene editing technologies have evolved rapidly, with CRISPR therapy now being an approved therapy for sickle cell disease [144]. Rapid improvement in gene-editing tool design, especially the optimisation of CRISPR-based tools, such as PE, BE, CRISPRa, and CRISPRi, has resulted in improved safety and reduced off-target effects, but these tools still require improved delivery capacity to the lung [32]. Despite advances in delivery strategies, their therapeutic potential in the lung remains limited due to the complex structure of the lung, delivery barriers, and organ-specific safety considerations. Next-generation vectors, including engineered AAV capsids and selective-organ targeting nanoparticles aim to enhance tropism, increase payload capacity, and reduce immunogenicity. Developing immune-evasive delivery systems is essential not only to enable delivery but for repeat dosing. Increasing evidence that epithelial metabolic state influences nanoparticle uptake also suggests future delivery systems may need to be tailored to disease-specific microenvironments.

Translating genome editing to the lung requires rigorous safety evaluation. Double-strand break-inducing nucleases can cause off-target cleavage, large deletions, or chromosomal rearrangements, risks that are particularly relevant for long-lived epithelial progenitors such as basal, club, and ATII cells. High-fidelity nucleases, transient editor expression, and DSB-free platforms offer safer alternatives to mitigate these risks but require assessment through comprehensive genomic integrity pipelines in relevant human systems. Human models must be able to evaluate editing efficiency, off-target activity, inflammatory responses, and tissue-level toxicity, in addition to disease-specific features such as mucus hypersecretion, fibrosis, and altered metabolism, enabling iterative optimisation before in vivo testing.

Therapeutics require the identification of disease-causing targets, and current gene-editing technologies are increasingly used to generate more representative lung disease models. These tools enable genetic manipulation of existing models, expanding the abilities of current ex vivo and in vitro models [17]. While monogenic causes of lung disease remain the main focus, epigenetic regulation and other factors continue to be elucidated [145]. Future more robust models must incorporate biological differences, including sex to improve their translational effects. Gene-editing tools are being designed and evolved to address these new avenues, such as Enhanced CRISPR–dCas9–based epigenetic editors [146].

Advances in editor engineering, delivery optimisation, and human-model validation are converging to move lung gene editing from conceptual feasibility toward clinical readiness. The coming decade is likely to see the first in vivo gene editing trials targeting airway and alveolar epithelium, marking a transformative shift in the treatment of chronic lung diseases.

## Figures and Tables

**Table 2:** Comparative table of human lung preclinical models for gene-editing.

Model	Key Cell Type	Key Advantages	Major Limitations
**Air–liquid interface (ALI)**	Airway epithelial cells	Physiologically relevant airway differentiation; accessible apical surface; ideal for aerosol/vector testing	Donor variability; limited lifespan; lacks immune, stromal, vascular components
**iPSC-derived lung cells**	AT1/AT2-like cells, airway basal cells, macrophages	Genetically defined; scalable; enables isogenic disease models	Developmental immaturity; differentiation variability; incomplete maturation
**3D Lung Organoids**	Epithelial cell, mesenchymal cells	Multicellular architecture; cell–cell interactions	Lack vascularisation, immune components; heterogeneity; limited delivery access
**Lung-on-a-chip**	Epithelial cells, endothelial cells, immune cells	Incorporates flow, stretch, barrier function	Technical complexity; low throughput; limited standardisation
**Precision cut lung slices (PCLS)**	All lung cell populations	Preserves architecture and microenvironment interactions	Limited viability; donor variability; diffusion barriers
**Ex vivo lung perfusion (EVLP)**	All lung cell populations	Organ-level physiology; real-time assessment	Limited organ availability; short experimental window; high cost/logistics

**Table 3:** Clinical trials using gene-editing as treatment for diseases associated with the lung registered on clinicaltrial.gov (accessed 17 September 2026).

NCT Number	Study Title	Conditions	Gene-Editing Context	Target Gene	Editing/Replacement Status	Target Cell/Organ	Delivery Platform/Route	Developmental Stage
NCT04990557	CRISPR/Cas9-modified Human T Cell (PD-1and ACE2 Knockout Engineered T Cells) for Inducing Long-term Immunity in COVID-19 Patients	COVID-19 respiratory infection	Systemic editing of ex vivo cells	Programmed cell death protein 1and ACE2 gene	Ex Vivo CRISPR Cas9 knockout and autologous transplant of edited T-cells	T-cell	IV infusion	Status unknown
NCT06389877	A Study to Evaluate the Safety and Efficacy of BEAM-302 in Adult Patients with Alpha-1 Antitrypsin Deficiency (AATD)	AATD (alpha-1 antitrypsin deficiency)	Systemic liver-targeted in vivo editing	PiZ variant SERPINA1	Adenine base-editor gene correction using a LNP delivery platform of an mRNA encoding adenine-base editor to correct the PiZ (E342K) variant	Liver	Lipid-based nanoparticle	Actively recruiting
NCT06892236	Preparation of IPSC for Cell Gene Editing for the Treatment of AATD	AATD (alpha-1 antitrypsin deficiency)	Systemic editing of ex vivo cells	SERPINA1	Base-editor correction	Induced pluripotent stem cells (iPSC) from blood and urine of patients differentiated to induced hepatocytes	Extracellular vesicles	Enrolling by invitation

## Data Availability

No new data were created or analysed in this study. Data sharing is not applicable to this article.
